# An autism spectrum disorder-related de novo mutation hotspot discovered in the GEF1 domain of Trio

**DOI:** 10.1038/s41467-017-00472-0

**Published:** 2017-09-19

**Authors:** Anastasiia Sadybekov, Chen Tian, Cosimo Arnesano, Vsevolod Katritch, Bruce E. Herring

**Affiliations:** 1 Department of Chemistry, University of Southern California, Los Angeles, CA 90089 USA; 2 Department of Biological Sciences, University of Southern California, Los Angeles, CA 90089 USA; 3 Neuroscience Graduate Program, University of Southern California, Los Angeles, CA 90089 USA; 4 Bridge Institute, University of Southern California, Los Angeles, CA 90089 USA

## Abstract

The Rho guanine nucleotide exchange factor (RhoGEF) Trio promotes actin polymerization by directly activating the small GTPase Rac1. Recent studies suggest that autism spectrum disorder (ASD)-related behavioral phenotypes in animal models of ASD can be produced by dysregulation of Rac1’s control of actin polymerization at glutamatergic synapses. Here, in humans, we discover a large cluster of ASD-related de novo mutations in Trio’s Rac1 activating domain, GEF1. Our study reveals that these mutations produce either hypofunctional or hyperfunctional forms of Trio in rodent neurons in vitro. In accordance with pathological increases or decreases in glutamatergic neurotransmission observed in animal models of ASD, we find that these mutations result in either reduced synaptic AMPA receptor expression or enhanced glutamatergic synaptogenesis. Together, our findings implicate both excessive and reduced Trio activity and the resulting synaptic dysfunction in ASD-related pathogenesis, and point to the Trio-Rac1 pathway at glutamatergic synapses as a possible key point of convergence of many ASD-related genes.

## Abstract (web summary)

Trio is a RhoGEF protein that promotes actin polymerization and is implicated in the regulation of glutamatergic synapses in autism spectrum disorder (ASD). Here the authors identify a large cluster of de novo mutations in the GEF1 domain of Trio in whole-exome sequencing data from individuals with ASD, and confirm that some of these mutations lead to glutamatergic dysregulation in vitro.

## Introduction

It is estimated that ~ 1% of the human population has a disorder on the Autism spectrum. Autism-spectrum disorders or ASDs are a range of neurodevelopmental disorders that are characterized by impaired social interaction, communication and restricted and repetitive behavior. About 40% of individuals with ASD also display some form of developmental delay while 70% display some level of intellectual disability. There is a strong genetic basis for ASD, and recent studies now establish an important role of germline de novo mutation in ASD-risk. De novo mutations identified in large exome sequencing studies have identified a number of new candidate ASD-risk genes and have ultimately led to a revised model for causation^1^. Over the past 10 years, evidence has also grown suggesting a convergence on altered regulation of glutamatergic synaptic development and function in ASD^2–4^. Disrupted synaptic actin modulation at glutamatergic synapses has been identified in well-established animal models of ASD, and in some cases has been identified as the underlying cause of ASD-related behavioral phenotypes in these models^5–7^. Rac1-mediated synaptic actin regulation in particular has been implicated in ASD and has been proposed as a likely point of convergence of many known ASD risk genes^5, 6, 8^.

We have recently discovered that the Rho guanine nucleotide exchange factor (RhoGEF) protein Trio, along with its paralog Kalirin, is required for glutamatergic neurotransmission^9^. Trio expression in the brain is highest in late prenatal/early postnatal development while Kalirin expression does not reach its peak until adolescence^10–12^. Multiple isoforms of Trio originating from a single gene are expressed in the brain^13^. Trio-9, for example, is the predominant isoform expressed in the cortex and hippocampus. Both Trio and Kalirin reside within the postsynaptic compartment of glutamatergic synapses referred to as dendritic spines^10, 14^. In spines, these two proteins regulate glutamatergic synapse function through the ability of their GEF1 domains to promote Rac1-dependent actin polymerization^9, 15, 16^. Furthermore, we have found that Trio and Kalirin are targets of CaMKII phosphorylation that are required for the induction of Long-Term Potentiation (LTP)^9^, the cellular process believed to underlie learning and memory. Thus, these proteins play fundamental roles in glutamatergic synapse regulation.

Here we identify a large cluster of ASD-related de novo mutations in the Rac1-activation domain of Trio, GEF1. The degree of mutational clustering that we find in Trio’s GEF1 domain and the computationally predicted impact of these ASD-related de novo mutations on Trio-Rac1 interactions suggest a strong association of Trio-Rac1 pathway dysregulation in ASD-related pathologies. Systematic examination of these mutations in Trio-9 reveals both hypomorphic and hypermorphic mutations that dramatically and bidirectionally affect Trio’s function and Trio’s influence on glutamatergic neurotransmission in hippocampal CA1 pyramidal neurons. Animal models of ASD exhibit pathological increases and decreases in glutamatergic neurotransmission^17–19^. Our study uncovers ASD-related missense mutations in a single synaptic Rac1-activating protein that can produce bidirectional alterations of glutamatergic neurotransmission and thus implicates both reduced and excessive Trio activity and the resulting synaptic dysfunction in ASD-related disease.

## Results

### ASD-related mutations in Trio

**Fig. 1:**
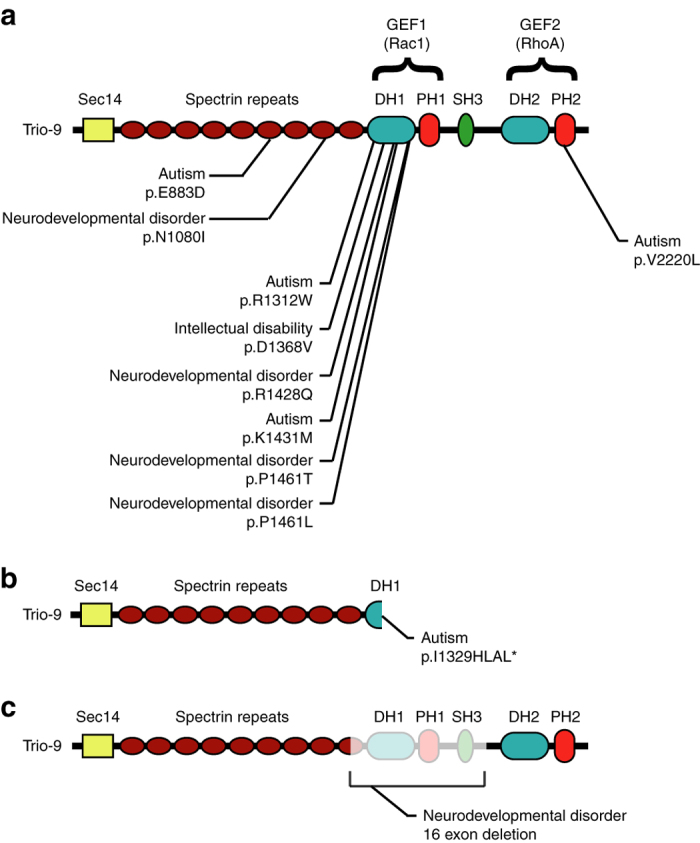
ASD-related de novo mutations in Trio. **a** Missense, **b** nonsense and **c** copy number variation mutations in Trio found in individuals with ASD-related disorders. The different protein domains are indicated, starting with the N-terminus: Sec14 domain, Spectrin repeats, GEF1 domain (composed of a Dbl homology domain (DH1) and a Pleckstrin homology domain (PH1)), Src homology 3 domain (SH3), and the GEF2 domain (composed of a Dbl homology domain (DH2) and a Pleckstrin homology domain (PH2)). For each mutation, the individual’s diagnosis is given along with information about the alteration of Trio’s amino acid sequence. Position of amino-acid mutations from NP_009049.2

Recent whole-exome sequencing studies have proved fruitful in uncovering risk-conferring variations, primarily by enumerating de novo variations, which are sufficiently rare that multiple mutations in a gene suggest a link to ASD. We queried several large databases of de novo mutations found specifically in individuals with ASD or ASD-related disorders (i.e. intellectual disability and neurodevelopmental disorders), looking for ASD-related mutations in either Trio or Kalirin^1, 20–24^. Together these studies included 4890 individuals with ASD-related disease. We found no ASD-related mutations in Kalirin but found a surprisingly large number of highly clustered ASD-related mutations in Trio’s GEF1 domain, specifically within the DH1 subdomain (GEF1/DH1) (Fig. 1 and Supplementary Table 1). Trio’s GEF1/DH1 subdomain binds directly to Rac1 and is essential for Trio’s ability to promote actin polymerization^25, 26^. The GEF1/DH1 subdomain of only 175 amino acids harbors six de novo missense mutations (Fig. 1a), as well as a single nucleotide deletion resulting in the formation of a stop codon inside Trio’s GEF1/DH1 subdomain (Fig. 1b). In addition, another individual was identified where a 16 exon deletion of the *TRIO* gene results in the removal of the entire GEF1 domain (Fig. 1c). We also found two missense mutations in Trio’s spectrin repeat region and one missense mutation in the PH subdomain of Trio’s GEF2 domain (Fig. 1a and Supplementary Table 1). None of these mutations were observed in the genomic sequences of family member controls^1, 20–24^. The number of observed de novo mutations in *TRIO* across these studies was on par with numbers of mutations in well-established ASD-related genes (e.g., *SCN2A*, *SYNGAP*, *SHANK2* and *SHANK3* and *MECP2*), however, *TRIO*’s GEF1/DH1 subdomain had the highest mutation rate per sequence base among these genes (Supplementary Table 2).

**Fig. 2:**
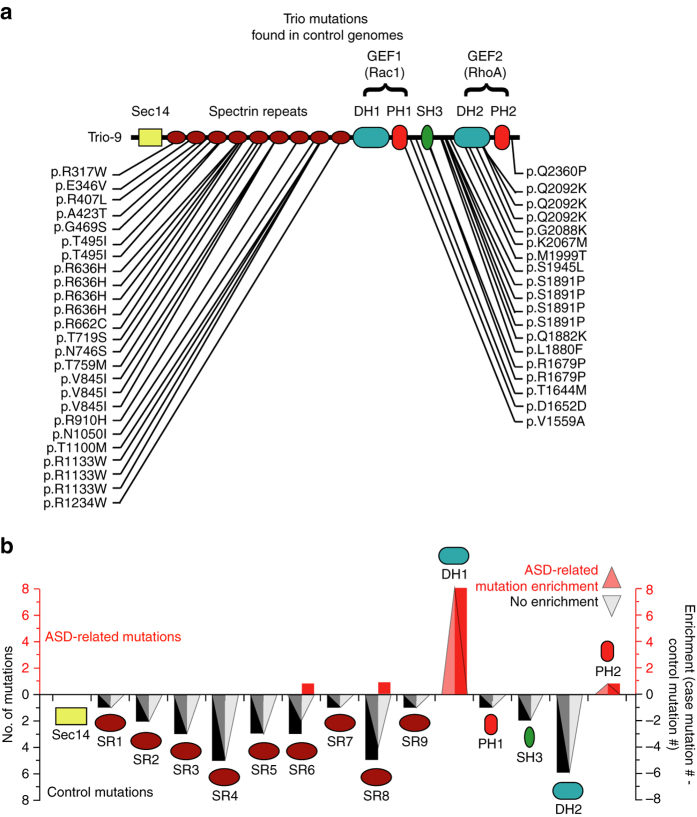
Trio mutations found in control and ASD-related genomes. **a** Trio-9 mutations found in control genomes (from De Rubeis et al., 2014^24^). **b** The graph shows the number of ASD-related (*red bars*) and control (*black bars*) mutations found in each domain of Trio-9 in individuals with ASD-related disorders and control individuals without ASD-related disorders, respectively. ASD-related Trio mutation enrichment in each domain of Trio was calculated by subtracting the number of control mutations from the number of ASD-related de novo mutations observed in each domain. *Transparent triangles* illustrate the presence (*red*), absence (*black*) and magnitude of ASD-related mutation enrichment in each domain

According to the recent Exome Aggregation Consortium (ExAC) analysis, based on 60,706 fully sequenced human genomes, *TRIO* is one of the top 60 most constrained human genes, suggesting a high functional importance. Trio has an exceptionally low rate of both missense mutations (*z* = 6.29) and loss of function (LoF) nonsense mutations (pLi = 1.), while having an average rate of synonymous mutations (*z* = 0.19)^27^. Even more striking is the constraint in the GEF1/DH1 domain. Remarkably, we observed no missense or loss of function (LoF) mutations in the GEF1/DH1 domain in 9,937 control genomes^24^ (Table 1 and Fig. 2a), thus indicating a strong enrichment of ASD-related mutations in this region (Fig. 2b). We also found no missense mutations in the GEF1/DH1 domain in the 1000 Genomes database^28^ (Supplementary Fig. 1a). Importantly, synonymous mutations listed in the 1000 Genomes database were present in this region (Supplementary Fig. 1b). Taken together, these data point to a strong enrichment of ASD-related de novo mutations in the GEF1/DH1 region of Trio.

**Table 1:** Case and control mutations found in Trio

Trio Domain:	Sec14	SR1	SR2	SR3	SR4	SR5	SR6	SR7	SR8	SR9	DH1	PH1	SH3	DH2	PH2
Case DN missense	0	0	0	0	0	0	1	0	1	0	6	0	0	0	1
Case DN LoF	0	0	0	0	0	0	0	0	0	0	2	0	0	0	0
Inherited missense	0	0	1	2	1	3	3	0	1	0	0	0	0	5	0
Inherited LoF	0	0	0	0	0	0	0	0	0	0	0	0	0	0	0
Control missense	0	1	1	1	4	0	0	1	4	1	0	1	2	1	0
Control LoF	0	0	0	0	0	0	0	0	0	0	0	0	0	0	0
Total Case	0	0	0	0	0	0	1	0	1	0	8	0	0	0	1
Total Control	0	1	2	3	5	3	3	1	5	1	0	1	2	6	0

The table shows the number of de novo (DN) missense and loss of function (LoF) mutations found in each domain of Trio-9 in patients with ASD-related diseases (Case) and individuals without an ASD-related disease (Control). Inherited mutations are mutations in Trio found in unaffected parents of offspring with ASD-related disease. ASD-related case mutations are from refs ^1, 20–24^. Control/inherited mutations are from ref. ^24^. Sec14 domain (Sec14), Spectrin repeats 1–9 (SR1-9), Dbl homology domain 1 (DH1), Pleckstrin homology domain 1 (PH1), Src homology 3 domain (SH3), Dbl homology domain 2 (DH2) and Pleckstrin homology domain 2 (PH2)

To assess significance of the Trio protein and Trio-GEF1/DH1 domain mutations in ASD-related disorders, we compared the expected number of de novo mutations to that observed. We used the mutational model developed by Samocha et al.^29^. The results of Samocha et al. were based on 1078 ASD cases and 151 cases of intellectual disability, and this study was able to identify *SCN2A* and *SYNGAP* as significant in ASD-related disorders. In the present study, our much larger case number (4890 for ASD-related disorders) and the large number of missense mutations identified in *TRIO* (11 ASD cases, as compared to 1.07 cases expected for the same number of individuals in the general population) dramatically improved the statistics, leading to a highly significant whole genome association of *TRIO* with ASD-like syndromes (*P*-value of < 1.96 × 10^−8^; Supplementary Table 3). We found an even stronger association with ASD-related syndromes for the Rac1 interacting GEF1/DH1 subdomain of *TRIO* (7 cases compared to 0.06 expected, *P*-value < 5.5 × 1^−13^; Supplementary Table 3).

### ASD mutations in Trio are predicted to alter Rac1 activation

**Fig. 3:**
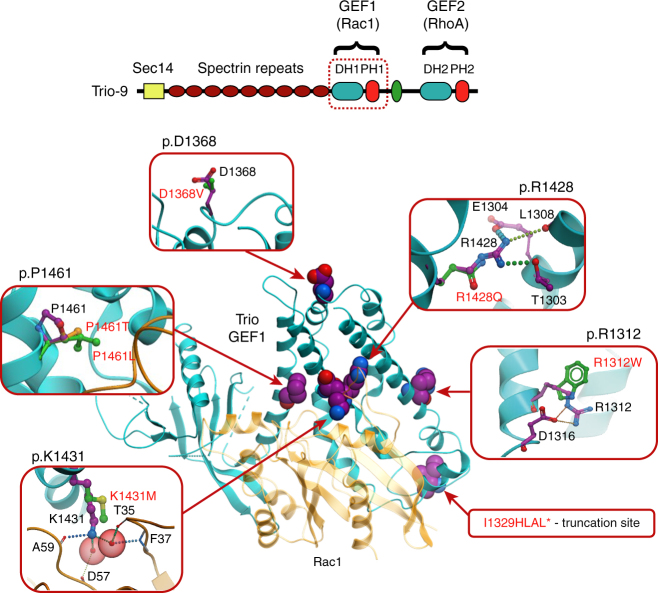
Predicted effects of ASD-related DH1 mutations on Rac1 activation. The GEF1 domain in the context of the entire protein is shown above with the GEF1 domain identified by a *dashed red square*. An overall view of the Trio-GEF1 and Rac1 complex structure is shown beneath, with the two interacting proteins shown as *blue* and *orange* cartoons, respectively (Protein Data Bank code 2NZ8). Amino-acid residues mutated in ASD-related disease are shown as *spheres* with carbon atoms colored *magenta*. Zoomed in inserts represent interactions between amino-acid residues in wild-type and mutant protein. Wild-type residues are labeled and shown in stick representation, with carbon atoms colored in *magenta*. Mutated amino acids are shown with carbon atoms colored *green* or *yellow*. Hydrogen bonds/salt bridges are shown as *dashed lines*. Water molecules involved in GEF1-Rac1 interactions are indicated by *transparent red spheres*

The clustered nature of Trio’s de novo mutations affecting the GEF1/DH1 subdomain suggests a connection to ASD. While the nonsense and copy number mutants (Fig. 1b, c) would apparently result in loss of Trio interactions with Rac1, the effects of the missense mutations (Fig. 1a) remain to be identified. We used structure-based modeling to assess the possibility that these mutations disrupt the ability of Trio’s GEF1/DH1 domain to activate Rac1. The structure of Trio’s GEF1 domain in complex with Rac1 has been solved^25^ (Fig. 3), which allows accurate conformational modeling and evaluation of mutation effects on stability and Rac1 binding (Fig. 3 and Table 2). Of the six missense mutations identified related to Trio’s GEF1/DH1 domain, five were predicted to disrupt GEF1-mediated Rac1 activation (Table 2). Two of these mutations (R1312W and R1428Q) were predicted to destabilize the 3D structure of Trio’s DH1 subdomain due to internal clashes and disruption of the internal hydrogen bonding network. The other three mutations (K1431M, P1461T and P1461L) were predicted to directly interfere with the ability of Trio’s DH1 subdomain to interact with Rac1 due to disruption of the intermolecular hydrogen bonding network (K1431M) or changing the backbone conformation of the protein at the interaction interface (P1461T and P1461L) (Fig. 3 and Table 2). All five of these mutations were found to be within 6 Å from the Rac1-interacting interface of the GEF1/DH1 subdomain. One notable exception among the GEF1/DH1 mutations, however, was D1368V, which is located away from the Rac1 interface, does not make any intramolecular interactions, and was predicted to not impact Rac1 activation. Here we find that this mutation has neurobiological consequences distinct from the other ASD-related GEF1/DH1 mutations characterized below. Modeling data along with available patient information were used to select specific ASD-related missense mutations for further study.

**Table 2:** Predictions of free energy change in Trio GEF1/DH1 domain stability and binding to Rac1

Mutation (protein level)	Mutation (DNA level)	ΔΔ*G*_bind_, kcal/mole	ΔΔ*G*_stability_, kcal/mole	Prediction
p.1149-1889 (deleted)	g.5:14380988-14463257(deleted)	—	—	Entire Rac1 interface is eliminated
p.I1329HLAL*	c.T3809_	—	—	Entire Rac1 interface is eliminated
p.R1312W	c.C3757T	0.9	3.1*	Disruption of the salt bridge to D1316
p.D1368V	c.A3926T	1	−0.3	*No significant effect on Rac1 binding*
p.R1428Q	c.G4106A	1.2	3.2*	Disruption of the salt bridge to E1304
				Disruption of the hydrogen bonds to T1303 and L1308
p.K1431M	c.A4115T	3.2*	−1.1	Disruption of the hydrogen bonds with Rac1 A59 backbone
				Disruption of water-mediated interactions with Rac1 T35, F37 and D57 backbone
p.P1461T	c.C4204A	2.2*	−1.3	Distortion of alpha-helix
p.P1461L	c.C4205T	7.8*	0.04	Distortion of alpha-helix

Predictions of free energy change in protein domain stability (∆∆*G*_stability_) and binding of Trio-GEF1 and Rac1 (∆∆*G*_bind_) for mutant proteins and expected effects of mutations on the ability of Trio to activate Rac1. Starred ∆∆ values indicate predicted inhibition of Rac1 activation. Position of amino-acid mutations from NP_009049.2 and position of corresponding nucleotide mutations from Human (GRCh37) or NM_007118.2

### Trio-9 I1329HLAL* expression inhibits synaptic function

**Fig. 4:**
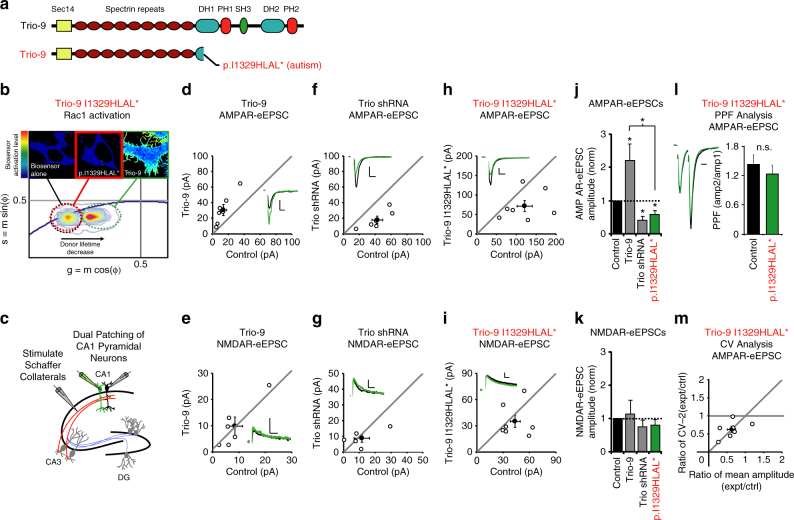
Trio-9 p.I1329HLAL* expression reduces the strength of glutamatergic synapses. **a** Illustrations of Trio-9 and Trio-9 I1329HLAL*. **b** Trio-9 I1329HLAL* inhibits Trio-9’s ability to activate Rac1. Representative FLIM color maps of HEK293 cells expressing the Rac1 biosensor, the biosensor and Trio-9 I1329HLAL* or the biosensor and Trio-9 are shown above. A *cropped phasor plot* for each condition is shown below. *Dashed ovals* identify the lifetime distribution for each condition. *Complete phasor plots* are shown in Supplementary Fig. 2. **c** Electrophysiological recording setup. **d**–**i** *Scatterplots* show eEPSC amplitudes for single pairs of control and transfected neurons (*open circles*). *Filled circles* show mean ± SEM. (*Insets*) Current traces from control (*black*) and transfected (*green*) neurons (*Scale bars*: 20 ms for AMPA, 50 ms for NMDA, 20 pA). **d** Trio-9 expression increased AMPAR-eEPSC amplitude (*n* = 7 pairs, *P < 0.05, Wilcoxon Signed Rank Test). **e** Trio-9 expression did not affect NMDAR-eEPSC amplitude (*n* = 6 pairs, *p* > 0.05, Wilcoxon Signed Rank Test). **f** Trio shRNA expression reduced AMPAR-eEPSC amplitude (*n* = 6 pairs, *P < 0.05, Wilcoxon Signed Rank Test). **g** Trio shRNA expression did not affect NMDAR-eEPSC amplitude (*n* = 5 pairs, *P* > 0.05, Wilcoxon Signed Rank Test). **h** Trio-9 I1329HLAL* expression reduced AMPAR-eEPSC amplitude (*n* = 7 pairs, *P < 0.05, Wilcoxon Signed Rank Test). **i** Trio-9 I1329HLAL* expression did not affect NMDAR-eEPSC amplitude (*n* = 7 pairs, *P* > 0.05, Wilcoxon Signed Rank Test). **j**, **k** Average eEPSC amplitudes (±SEM) of neurons expressing Trio-9, Trio shRNA and Trio-9 I1329HLAL* normalized to their respective average control eEPSC amplitudes. A Wilcoxon Rank Sum Test was used to compare across independent conditions, (i.e., Trio-9 and Trio-9 I1329HLAL* in **j**, *P < 0.05). **l** Mean ± SEM paired-pulse facilitation (PPF) ratios for Trio-9 I1329HLAL* expressing and paired control neurons (*n* = 6 pairs, *P* > 0.05, Student’s t-test). Peak 1-scaled current traces from control (*black*) and transfected (*green*) neurons (*Scale bar*: 20 ms). n.s., not significant. **m** CV analysis of AMPAR-eEPSCs from pairs of control/Trio-9 I1329HLAL* neurons. CV^−2^ is graphed against ratio of mean amplitude within each pair (open circles). *Filled circle* shows mean ± SEM. (*n* = 7 pairs)

The first mutation we examined was Trio-9 I1329HLAL* (Fig. 4a). This nonsense mutation, found in an individual diagnosed with ASD, results in truncation of Trio inside its GEF1/DH1 subdomain and eliminates the Rac1 interface of this region. To examine the impact this mutation has on the ability of Trio-9 to activate Rac1 we used a recently developed fluorescence resonance energy transfer (FRET)-based biosensor that measures the ability of RhoGEFs to activate Rac1^30^. Initially we transfected HEK293 cells with this Rac1 biosensor and wild-type Trio-9. Using fluorescence lifetime imaging (FLIM) to detect FRET, we found that HEK293 cells co-expressing wild-type Trio-9 and this biosensor exhibited a much higher FRET signal relative to cells expressing the biosensor alone (Fig. 4b and Supplementary Fig. 2a). In the representative cell color map images, colors range from blue (low Rac1 biosensor activation) to red (high Rac1 biosensor activation). We observed a larger population of quenched donor molecules in the pixels located toward the cell perimeters compared to the inner region of the cells, indicating a higher level of Rac1 activity at these locations. In contrast to wild-type Trio-9, the FRET signal observed in HEK293 cells co-expressing the biosensor and Trio-9 I1329HLAL* was very low (Fig. 4b and Supplementary Fig. 2b). The near complete lack of Rac1 biosensor activation observed in Trio-9 I1329HLAL* expressing cells looked very similar to those cells that were transfected with the biosensor alone (Fig. 4b and Supplementary Fig. 2b). Thus, Trio-9 I1329HLAL* dramatically reduces Trio-9-mediated Rac1 activation.

We were then interested in the impact Trio-9 I1329HLAL* expression has on glutamatergic synapse function. First, we used biolistic transfection to express wild-type Trio-9 in CA1 pyramidal neurons of organotypic rat hippocampal slice cultures. 6 days after transfection recordings of AMPA receptor and NMDA receptor-evoked excitatory postsynaptic currents (AMPAR and NMDAR-eEPSCs) were made from fluorescent transfected neurons and neighboring untransfected control neurons simultaneously during stimulation of Schaffer collaterals (Fig. 4c). This approach permits a pair-wise, internally controlled comparison of the consequences of the genetic manipulation that is not confounded by fluorophore expression (Supplementary Fig. 3). As reported previously^9^, we found that expression of Trio-9 resulted in a selective increase in AMPAR-eEPSC amplitude (Fig. 4d, e, j, k). Conversely, we found that knocking down Trio expression in these neurons using a short hairpin RNA (shRNA) against Trio resulted in a selective reduction in AMPAR-eEPSC amplitude (Fig. 4f, g, j, k). Recombinant Trio-9 rescues synaptic phenotypes resulting from Trio knockdown^9^. We then expressed Trio-9 I1329HLAL* for 6 days in CA1 pyramidal neurons. In contrast to the increase in AMPAR-eEPSC amplitude we observed with Trio-9, Trio-9 I1329HLAL* resulted in a ~40% decrease in AMPAR-eEPSC amplitude (Fig. 4h, j). Trio-9 I1329HLAL* expression had no effect on NMDAR-eEPSC amplitude (Fig. 4i, k). This phenotype was remarkably similar to that observed with the Trio shRNA. Paired-pulse facilitation (PPF) was also unchanged by postsynaptic expression of Trio-9 I1329HLAL* indicating that the expression of this mutant did not affect presynaptic glutamate release (Fig. 4l).

The selective reduction we observe in AMPAR-eEPSC amplitude produced by Trio-9 I1329HLAL* expression could be due to either a reduction in the number of AMPARs at all glutamatergic synapses or an increase in the number of synapses that lack AMPARs entirely. To determine which of these possibilities had occurred we performed coefficient of variation (CV) analysis on eEPSC current amplitudes (Fig. 4m and Supplementary Fig. 4). CV analysis can be used to determine the quantal parameters of glutamatergic transmission in control and transfected neurons^31–35^. By comparing the normalized variance in eEPSC amplitudes from two neurons receiving the same stimulus, it is possible to determine relative quantal size and quantal content. Changes in quantal size precisely change both the mean eEPSC and the variance such that the normalized ratio of mean^2^/variance, also known as coefficient of variation (or CV), remains constant. Changes in quantal size cause the marker of the mean to fall on the horizontal line seen in Fig. 4m and in this case, would denote a change in the number of glutamatergic receptors at each synapse. In contrast, changes in quantal content will produce proportional changes of equal magnitude in CV that cause the marker of the mean to fall on the diagonal line and indicate a change in the number of functional synapses. We observed equal reductions in CV and AMPAR-eEPSC amplitude in neurons expressing Trio-9 I1329HLAL* (Fig. 4m). This result suggests a reduction in quantal content rather than quantal size as responsible for this reduction of AMPAR-eEPSC amplitude. Given that Trio-9 I1329HLAL* expression did not alter NMDAR-eEPSC amplitude or PPF, this finding indicates that Trio-9 I1329HLAL* expression increases the number of AMPAR-less or “silent” synapses. As with wild-type Trio-9, variation in NMDAR-eEPSC amplitudes in Trio-9 I1329HLAL* expressing neurons was due to variation in quantal content (Supplementary Fig. 4a, b). Together these data suggest that Trio-9 I1329HLAL* competes with wild-type Trio, effectively lowers the concentration of functional Trio at glutamatergic synapses and results in a selective and potentially pathogenic reduction in synaptic AMPAR function.

### Trio-9 K1431M expression inhibits synaptic function

**Fig. 5:**
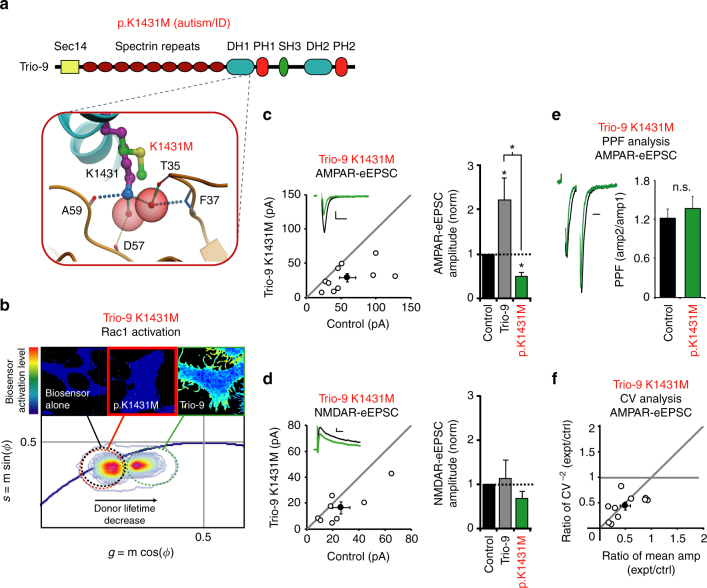
Trio-9 p.K1431M expression reduces the strength of glutamatergic synapses. **a** Predicted alteration to interaction between Trio-9 and Rac1 resulting from Trio-9 K1431M. ID, intellectual disability. **b** Trio-9 K1431M inhibits Trio-9’s ability to activate Rac1. Representative FLIM color maps of HEK293 cells expressing the Rac1 biosensor alone, the biosensor and Trio-9 K1431M or the biosensor and Trio-9 are shown above. A *cropped phasor plot* for each condition is shown below. *Dashed ovals* identify the lifetime distribution for each condition. *Complete phasor plots* are shown in Supplementary Figure 2. **c**, **d** *Scatterplots* show eEPSC amplitudes for single pairs of control and transfected neurons (*open circles*). *Filled circles* show mean ± SEM. (*Insets*) Current traces from control (*black*) and transfected (*green*) neurons (*Scale bars*: 20 ms for AMPA, 50 ms for NMDA, 20 pA). *Bar graphs* show the average eEPSC amplitudes (±SEM) of neurons expressing Trio-9 (in *gray*, from Fig. 4j, k) and Trio-9 K1431M normalized to their respective average control eEPSC amplitudes. In the *bar graphs* a Wilcoxon Rank Sum Test was used to compare across independent conditions (i.e., Trio-9 and Trio-9 K1431M in **c**, *P < 0.05). **c** Trio-9 K1431M expression reduced AMPAR-eEPSC amplitude (*n* = 10 pairs, *P < 0.05, Wilcoxon Signed Rank Test). **d** Trio-9 K1431M expression did not affect NMDAR-eEPSC amplitude (*n* = 8 pairs, *P* > 0.05, Wilcoxon Signed Rank Test). **e** Mean ± SEM paired-pulse facilitation (PPF) ratios for Trio-9 K1431M expressing and paired control neurons (*n* = 5 pairs, *P* > 0.05, Student’s *t*-test). Peak 1-scaled current traces from control (*black*) and transfected (*green*) neurons (*Scale bar*: 20 ms). n.s., not significant. **f** CV analysis of AMPAR-eEPSCs from pairs of control/Trio-9 K1431M neurons. CV^−2^ graphed against ratio of mean amplitude within each pair (*open circles*). *Filled circle* shows mean ± SEM. (*n* = 10 pairs)

We then examined the Trio missense mutation, Trio-9 K1431M. The individual harboring this mutation displayed severe autistic symptoms and intellectual disability. It is of note that a previous study using alanine-scanning mutations to identify important residues in Trio’s GEF1 region identified this residue along with R1428 as critical for Rac1 activation^36^. Our modeling of the K1431M mutation predicted a disruption to a number of hydrogen and water-mediated interactions resulting in a reduced affinity between Trio’s GEF1/DH1 domain and Rac1 (Fig. 3 and Table 2). FLIM analysis revealed that Trio-9 K1431M, like Trio-9 I1329HLAL*, severely reduced Trio-9’s ability to activate the Rac1 biosensor in HEK293 cells (Fig. 5b). Thus, as predicted, Rac1 activation was severely reduced by this mutation. We then expressed Trio-9 K1431M in CA1 pyramidal neurons and found that Trio-9 K1431M phenocopied Trio-9 I1329HLAL*. Trio-9 K1431M produced a ~50% reduction in AMPAR-eEPSC amplitude (Fig. 5c) that is also likely caused by an increase in silent synapses given that a reduction in quantal content occurred with no change in NMDAR-eEPSC amplitude or change in PPF (Fig. 5d–f). As with wild-type Trio-9, variation in NMDAR-eEPSC amplitudes in Trio-9 K1431M expressing neurons was due to variation in quantal content (Supplementary Fig. 4c). Together these data show that Trio-9 K1431M, like Trio-9 I1329HLAL*, likely competes with endogenous, wild-type Trio at synapses and results in a reduction in synaptic AMPAR function. Additionally, we found that a missense mutation in Trio’s GEF1 domain that was identified in an individual without ASD (Trio-9 S1575N)^28^ had no effect on Trio-9’s ability to increase AMPAR-eEPSC amplitude in CA1 pyramidal neurons (Supplementary Fig. 1a and 5).

### Trio-9 P1461T expression inhibits synaptic function

**Fig. 6:**
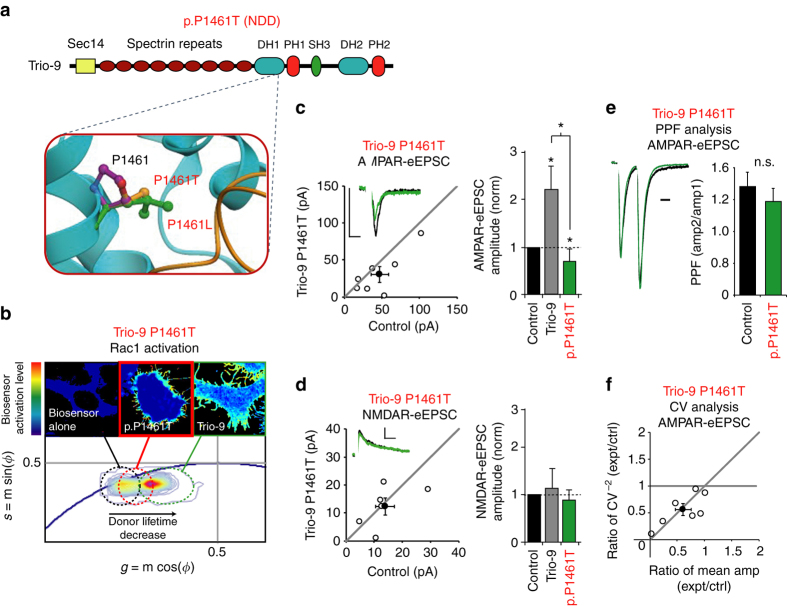
Trio-9 p.P1461T expression reduces the strength of glutamatergic synapses. **a** Predicted alteration to Trio’s GEF1 domain resulting from Trio-9 P1461T. NDD, neurodevelopmental disorder. **b** Trio-9 P1461T inhibits Trio-9’s ability to activate Rac1. Representative FLIM color maps of HEK293 cells expressing the Rac1 biosensor alone, the biosensor and Trio-9 P1461T or the biosensor and Trio-9 are shown above. A *cropped phasor plot* for each condition is shown below. *Dashed ovals* identify the lifetime distribution for each condition. *Complete phasor plots* are shown in Supplementary Fig. 2. **c**, **d** *Scatterplots* show eEPSC amplitudes for single pairs of control and transfected neurons (*open circles*). *Filled circles* show mean ± SEM. (*Insets*) Current traces from control (*black*) and transfected (*green*) neurons (*Scale bars*: 20 ms for AMPA, 50 ms for NMDA, 20 pA). *Bar graphs* show the average eEPSC amplitudes (±SEM) of neurons expressing Trio-9 (in *gray*, from Fig. 4j, k) and Trio-9 P1461T normalized to their respective average control eEPSC amplitudes. In the *bar graphs* a Wilcoxon Rank Sum Test was used to compare across independent conditions (i.e., Trio-9 and Trio-9 P1461T in **c**, *P < 0.05). **c** Trio-9 P1461T expression reduced AMPAR-eEPSC amplitude (*n* = 7 pairs, *P < 0.05, Wilcoxon Signed Rank Test). **d** Trio-9 P1461T expression did not affect NMDAR-eEPSC amplitude (*n* = 6 pairs, *P* > 0.05, Wilcoxon Signed Rank Test). **e** Mean ± SEM paired-pulse facilitation (PPF) ratios for Trio-9 P1461T expressing and paired control neurons (*n* = 5 pairs, *P* > 0.05, Student’s *t-*test). Peak 1-scaled current traces from control (*black*) and transfected (*green*) neurons (*Scale bar*: 20 ms). n.s., not significant. **f** CV analysis of AMPAR-eEPSCs from pairs of control/Trio-9 P1461T neurons. CV^−2^ graphed against ratio of mean amplitude within each pair (*open circles*). *Filled circle* shows mean ± SEM. (*n* = 7 pairs)

Additional ASD-related missense mutations predicted to impair the interaction between Trio’s GEF1/DH1 domain and Rac1 were two mutations in the P1461 position, P1461T and P1461L (Fig. 3, Table 2 and Fig. 6a). Both individuals had severe neurodevelopmental disorders. de novo mutations in the same protein residue position in two unrelated individuals with similar disorders strongly suggests that such mutations contribute to the development of the disorder. As both mutations were predicted to destabilize Rac1 binding, we examined the impact P1461T has on Trio-9 function. While we did observe some FRET in HEK293 cells expressing Trio-9 P1461T and the Rac1 biosensor, the level of biosensor activation in these cells was lower than those cells expressing wild-type Trio-9 (Fig. 6b). Thus, this mutation lowers Trio’s ability to activate Rac1, albeit not to the extent observed with Trio-9 I1329HLAL* and Trio-9 K1431M. Even though this mutation resulted in a less severe inhibition of Trio-9’s ability to activate Rac1, expression of Trio-9 P1461T in neurons led to a synaptic phenotype that was similar to Trio-9 I1329HLAL* and Trio-9 K1431M. Like Trio-9 I1329HLAL* and Trio-9 K1431M, Trio-9 P1461T expression in hippocampal CA1 pyramidal neurons reduced AMPAR-eEPSC amplitude (Fig. 6c) by reducing quantal content without affecting NMDAR-eEPSC amplitude or PPF (Fig. 6d–f). As with wild-type Trio-9, variation in NMDAR-eEPSC amplitudes in Trio-9 P1461T expressing neurons was due to variation in quantal content (Supplementary Fig. 4d). Together, these findings again suggest an increase in silent synapse number. Thus, Trio-9 I1329HLAL*, Trio-9 K1431M and Trio-9 P1461T expression in neurons impact glutamatergic neurotransmission in a similar manner.

### Trio-9 D1368V results in Trio hyperfunction

**Fig. 7:**
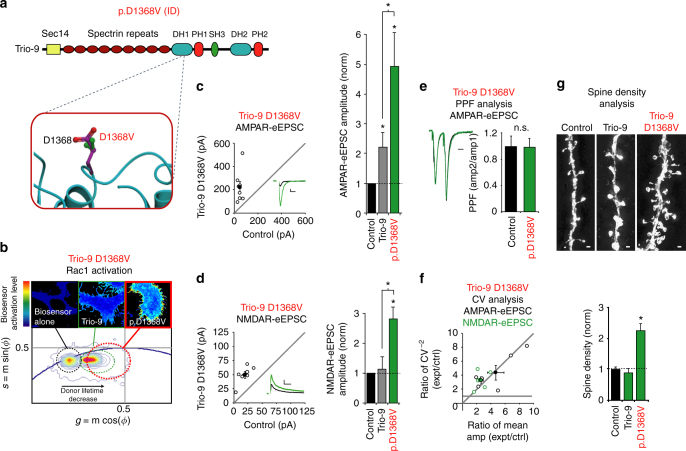
Trio-9 p.D1368V produces a hyperfunctional synaptogenic form of Trio. **a** Predicted alteration to Trio’s GEF1 domain produced by Trio-9 D1368V. ID, intellectual disability. **b** Trio-9 D1368V increases Trio-9’s ability to activate Rac1. Representative FLIM color maps of HEK293 cells expressing the Rac1 biosensor alone, the biosensor and Trio-9 D1368V or the biosensor and Trio-9 are shown above. A *cropped phasor plot* for each condition is shown below. *Dashed ovals* identify the lifetime distribution for each condition. *Complete phasor plots* are shown in Supplementary Fig. 2. **c**, **d** *Scatterplots* show amplitudes of eEPSCs for single pairs of control and transfected neurons (*open circles*). *Filled circles* show mean ± SEM. (*Insets*) Current traces from control (*black*) and transfected (*green*) neurons (*Scale bars*: 20 ms for AMPA, 50 ms for NMDA, 20 pA). *Bar graphs* show the average eEPSC amplitudes (±SEM) of neurons expressing Trio-9 (in *gray*, from Fig. 4j, k) and Trio-9 D1368V normalized to their respective average control eEPSC amplitudes. In the *bar graphs* a Wilcoxon Rank Sum Test was used to compare across independent conditions (i.e., Trio-9 and Trio-9 D1368V in **c**, **d** , *P < 0.05). **c** Trio-9 D1368V expression increased AMPAR-eEPSC amplitude (*P < 0.05, Wilcoxon Signed Rank Test). **d** Trio-9 D1368V expression increased NMDAR-eEPSC amplitude (*n* = 6 pairs, *P < 0.05, Wilcoxon Signed Rank Test). **e** Mean ± SEM paired-pulse facilitation (PPF) ratios for Trio-9 D1368V expressing and paired control neurons (*P* > 0.05, Student’s *t*-test). Peak 1-scaled current traces from control (*black*) and transfected (*green*) neurons (*Scale bar*: 20 ms). n.s., not significant. **f** CV analysis of AMPAR and NMDAR-eEPSCs from pairs of control/Trio-9 D1368V neurons. CV^−2^ graphed against ratio of mean amplitude within each pair (*open circles*). *Filled circle* shows mean ± SEM. (AMPAR-eEPSCs, *n* = 6 pairs; NMDAR-eEPSCs, *n* = 6 pairs). **g** Trio-9 D1368V expression increased dendritic spine density. Representative dendritic spine images from neurons transfected with GFP (control), wild-type Trio-9 or Trio-9 D1368V are shown above (*Scale bars*: 1 μm). The *bar graph* below shows average spine density (mean ± SEM) of neurons expressing wild-type Trio-9 or Trio-9 D1368V normalized and compared to GFP expressing control neurons (control, *n* = 6; Trio-9, *n* = 5; Trio-9 D1368V, *n* = 5, *P < 0.05, Student’s *t*-test)

Lastly, we examined the impact of the GEF1/DH1 D1368V missense mutation found in an individual with severe intellectual disability. Unlike all the other ASD-related GEF1/DH1 missense mutations that have been identified, the D1368 residue is highly exposed to solvent, and is located in the GEF1/DH1 subdomain far away from the Rac1 binding interface (Fig. 3 and Fig. 7a). Indeed, our modeling of this mutation did not predict any direct impact on the stability of Trio’s GEF1 domain or its ability to activate Rac1 (Fig. 3 and Table 2). Therefore, we were curious if this mutation has any impact on Trio-9 function. We observed higher levels of Rac1 biosensor activation in cells expressing Trio-9 D1368V compared to wild-type Trio-9 (Fig. 7b); this unexpected finding suggested that Trio-9 D1368V increases Trio’s ability to activate Rac1. Remarkably, we found that expression of Trio-9 D1368V in CA1 pyramidal neurons resulted in a dramatic increase in AMPAR-eEPSC amplitude that was about twice that seen with wild-type Trio-9 (Fig. 7c). Trio-9 D1368V expression in CA1 pyramidal neurons also led to an unexpected increase in NMDAR-eEPSC amplitude (Fig. 7d). PPF was not affected by this mutation (Fig. 7e). To determine whether the observed enhancements of AMPAR and NMDAR-mediated transmission were produced by an increase in the number of glutamatergic synapses or an increase in the expression of AMPARs and NMDARs at existing synapses we again performed CV analysis. We found that increases in both AMPAR and NMDAR-eEPSC amplitude likely result from changes in quantal content rather than quantal size (Fig. 7f). This finding suggested that Trio-9 D1368V-mediated enhancement of AMPAR and NMDAR function is produced by an increase in the number of functional glutamatergic synapses. We reasoned that if Trio-9 D1368V expression leads to the formation of additional glutamatergic synapses, then a corresponding increase in dendritic spine density should be observed in these neurons. We used Structured Illumination Microscopy (SIM) to obtain super resolution images of dendritic spines from neurons expressing GFP, wild-type Trio-9 or Trio-9 D1368V. While wild-type Trio-9 expression had no effect on dendritic spine density, expression of Trio-9 D1368V resulted in a greater than twofold increase in the density of dendritic spines compared to GFP expressing control neurons (Fig. 7g). Together these data reveal that the Trio-9 D1368V mutation is a hypermorphic mutation resulting in a hyperfunctioning synaptogenic form of Trio that when expressed in neurons is capable of producing an abnormally high number of glutamatergic synapses.

## Discussion

Here we find a hotspot of de novo ASD-related mutations clustered specifically in Trio’s GEF1/DH1 subdomain (*P*-value < 5.5 × 10^−13^). Such a high number of potentially function-disrupting mutations suggests that these mutations contribute to the development of ASD-related disorders. In support of this hypothesis, we found that every GEF1/DH1 ASD-related mutation we tested greatly altered Trio’s influence on glutamatergic synapse function in the hippocampus. We identify both hypomorphic and hypermorphic ASD-related mutations in Trio, thus implicating reduced as well as excessive Trio activity in ASD-related pathogenesis. Trio’s involvement in ASD-related disease is further reinforced by recent human genome analysis studies that rank *TRIO* among their top ASD-candidate genes^37^.

Specific association of Trio mutations with ASD is supported by analysis of its close paralog, Kalirin, which has a unique developmental expression profile. No ASD-associated missense mutations were observed in Kalirin, despite playing a similar role in glutamatergic synapse regulation^9^. Trio is highly expressed in early postnatal development and decreases with age^10^. In contrast, Kalirin expression does not peak until adolescence^11, 12^. Remarkably, Kalirin function has been implicated in later onset neuropsychiatric disorders like schizophrenia and Alzheimer’s disease^38–41^. A missense mutation in Kalirin’s GEF1/DH1 domain that inhibited Rac1 activation has also been found in an individual with schizophrenia^39^. Thus, the expression profiles of Trio and Kalirin appear to match the age of onset of the diseases in which they are implicated, and suggest that disruption of Rac1-mediated synaptic regulation at different time points of brain development gives rise to distinct brain-related diseases.

Dysregulation of glutamatergic transmission is believed to underlie the development of ASD, and existing animal models of ASD display varied glutamatergic synapse phenotypes^2^. *MeCP2* ^*Tg1*^ and *MET* knockout mice, for example, display enhanced glutamatergic neurotransmission while *SHANK3* knockout and *MeCP2* ^*null/y*^ mice display deficits in glutamatergic signaling^17–19^. While the mechanisms underlying the glutamatergic synapse phenotypes observed in these animals are unclear, such data suggest that alteration of glutamatergic synaptic strength in either direction during synapse formation in early postnatal development likely results in global network changes contributing to ASD-related disease. By regulating actin filament formation through its GEF1 domain, Trio regulates glutamatergic synapse function via its effect on synaptic structure. Here we find that ASD-related missense mutations in Trio give rise to both decreased and increased glutamatergic synaptic function resulting from an increase in silent synapse number or enhanced synaptogenic capability, respectively. Thus, our data suggest that pathological repercussions result from both Trio hypofunction and Trio hyperfunction during development. Furthermore, to the best of our knowledge, our study is the first to show that ASD-related missense mutations in a single protein can produce bidirectional alterations of glutamatergic synapse function.

Our modeling and FLIM analysis identify the majority of Trio’s ASD-related GEF1/DH1 mutations as detrimental to Trio’s ability to activate Rac1. We also find that expression of several hypomorphic ASD-related Trio mutants in CA1 pyramidal neurons leads to selective reductions in AMPAR-eEPSC amplitude. While the exact mechanism of ASD-related hypomorphic mutations reducing synaptic AMPAR expression is unknown, the likely explanation is that the expressed hypomorphic Trio mutants compete with wild-type Trio for access to synapses and effectively lower the synaptic concentration of functional Trio. It is additionally possible that these hypomorphic Trio proteins that lack functional GEF1 domains compete with wild-type Trio for association with key synaptic regulatory protein complexes. Alternatively, expressed hypomorphic Trio mutants may target and promote degradation of wild-type Trio through some unknown mechanism. Such antagonistic relationships between hypomorphic and wild-type forms of Trio are important because the individuals that harbor the de novo Trio mutations described in this study are heterozygous for these mutations. Given that the majority of ASD-related mutations in Trio’s GEF1/DH1 domain potentially work against wild-type Trio function, these individuals are expected to have a level of Trio function below what would occur with a simple deletion of one copy of the gene. Alternatively, it is possible that these ASD-related Trio mutations are expressed at lower levels compared to wild-type Trio or are mis-targeted and thus do not reach synapses. However, we would presume that if these were the primary effects of these mutations, overexpression of such mutants would produce either smaller increases in AMPAR-eEPSC amplitude or simply produce no effect on synaptic transmission relative to control neurons. What we observe with hypomorphic Trio mutant expression are similar reductions in AMPAR-eEPSC amplitude that are consistent with these mutants inhibiting wild-type Trio function through either competition or degradation. Going forward, knock-in mice heterozygous for these hypomorphic ASD-related Trio mutations will be necessary to precisely identify ASD-related behavioral phenotypes that arise when Trio function is potentially in-between what occurs in Trio knockout mice and mice with a single *TRIO* allele. These mice will also be very useful in examining the impact ASD-related Trio mutations have on synaptic function at different time points in development, and may aid in identifying novel ways in which these mutations impact glutamatergic synapse function. For example, Trio’s recent implication in neurotransmitter release at the *Drosophila* neuromuscular junction now justifies an investigation into possible presynaptic roles for Trio at mammalian synapses^42^.

In this study, we also identify a hypermorphic missense mutation in Trio’s GEF1 domain that produces a dramatic enhancement of Trio activity. We find that Trio-9 D1368V expression results in an enhancement of AMPAR-mediated transmission that is twice that produced by wild-type Trio. Trio-9 D1368V also results in a substantial enhancement of NMDAR-mediated currents, which are not affected by wild-type Trio. CV and dendritic spine analysis identify an increase in the number of functional synapses as being responsible for this observed increase in glutamatergic neurotransmission. This finding is akin to reports of increased glutamatergic neurotransmission in several animal models of ASD/ID and observed increases in the number of glutamatergic synapses in a number of individuals with ASD^17, 18, 43^. Interestingly, this mutation is the only ASD-related mutation identified in our study that was predicted to not directly influence Trio’s ability to activate Rac1. Furthermore, this residue is highly exposed to solvent on a region of the GEF1/DH1 subdomain that is pointing away from the interface with Rac1. One possibility is that D1368V destabilizes autoinhibitory interactions that occur in Trio. Such intramolecular interactions have been proposed for Kalirin and other RhoGEFs^44, 45^. Another possibility is that this site is involved in the binding of allosteric inhibitors of GEF domains. TRIOBP, for example, has been shown to bind directly to Trio’s GEF1 domain and inhibit the ability of Trio to activate Rac1^46^. Alternatively, it is possible that this mutation increases Trio expression levels (e.g., through disruption of an E3 ligase binding substrate). Additional study will be needed to elucidate the mechanism by which this mutation results in enhanced Trio function.

Disruptions in synaptic Rac1-mediated actin regulation have recently been found to underlie the development of ASD-related behavioral phenotypes in well-established animal models of ASD-related diseases^5–7^. A recent study also identified Rac1 as a likely point of convergence of a number of ASD risk genes^8^. Here we identify the Rac1 activation domain of the synaptic actin regulatory protein, Trio, as a hotspot for ASD-related de novo mutations. Because dysregulation of glutamatergic neurotransmission is believed to underlie the behavioral phenotypes in many animal models of ASD and because proteins downstream of Trio activity have not been found to harbor substantial ASD-related mutations, it is tempting to speculate that altered Trio function represents a major point of convergence of a number of factors that give rise to ASD. Going forward it will be necessary to determine the prevalence of altered Trio function in individuals with ASD and establish whether Trio-related therapies can be applied to reverse ASD-related behavioral phenotypes in appreciable numbers of individuals with this disease.

## Methods

### Mutation modeling

The effect of mutations on stability and binding were predicted using the high-resolution crystal structure of Trio GEF1 complex with Rac1 (PDB code 2NZ8). Calculations were performed using ICM molecular modeling software (Molsoft LLC). Energy optimization of mutant protein conformation was performed using biased probability Monte Carlo algorithm. The free energy change in protein stability ∆∆*G*_*stability*_ (1) and protein binding ∆∆*G*_*bind*_ (2) was then calculated as a difference in folding or binding free energies of mutant and wild-type protein:

1 $$\Delta \Delta {G_{\rm{stability}}} = \left( {\Delta G_{{\rm{folded}}}^{{\rm{mutant}}} - \Delta G_{{\rm{unfolded}}}^{{\rm{mutant}}}} \right) - \left( {\Delta G_{{\rm{folded}}}^{{\rm{WT}}} - \Delta G_{{\rm{unfolded}}}^{{\rm{WT}}}} \right)$$

2 $$\Delta \Delta {G_{{\rm{bind}}}} = \Delta G_{{\rm{bind}}}^{{\rm{mutant}}} - \Delta G_{{\rm{bind}}}^{{\rm{WT}}}$$

Mutations with either *∆∆G*_bind_ > 2 or *∆∆G*_stability_ > 2 were predicted as disruptive for Trio activation of Rac1.

### Experimental constructs

Human Trio-9 (or Trio-9s in ref. ^13^) was generated from a Trio-FL cDNA generously provided by Dr. Betty A. Eipper (University of Connecticut). ASD-related Trio mutations were made from Trio-9 cDNA using overlap-extension PCR followed by In-fusion cloning (Clontech). All plasmids were confirmed by DNA sequencing. Trio-9 cDNAs were cloned into a pCAGGs vector containing IRES-mCherry. A pFUGW vector expressing only GFP was co-expressed with pCAGG-IRES-mCherry constructs to enhance identification of transfected neurons and was used as a control vector for spine imaging. The Rac1 biosensor construct was within a pTriEx-HisMyc backbone, has been described previously in ref. ^30^ and was generously provided by Dr. Jaap D. van Buul (Sanquin).

### HEK293 cell transfection

HEK293T cells (ATCC) were cultured in DMEM with 10% FBS in a 37 °C incubator supplied with 5% CO_2_. Cells were plated onto 35 mm glass bottom plates coated with Matrigel. Cells were grown until 50% confluence before being transfected with the Trio-9 expression constructs together with the Rac1 biosensor using FuGENE HD Transfection Reagent. A Trio-9/Rac1 biosensor DNA ratio of 1:1 was used. Plated cells were incubated in the transfection mixture for 16 h, and then replaced with fresh media. HEK293 experiments were conducted ~20 h after transfection.

### Fluorescence lifetime imaging (FLIM)

Fluorescence lifetime images were acquired from HEK293 cells with a Zeiss LSM-780 inverted microscope coupled to a Ti:Sapphire laser system (Coherent Chameleon Ultra II, 80 fs pulses with repetition rate of 80 MHz) and an ISS A320 FastFLIM^47, 48^. A 40X 1.1 NA water immersion objective (Zeiss Korr C-Apochromat) optimized for 2-P imaging was used. For image acquisition, the following settings were used: image size of 256 × 256 pixels and scan speed of 12.6 μs/pixel. A short pass dichroic filter (760 nm) was used to separate the fluorescence signal from the laser light. For the acquisition of FLIM images, fluorescence light was separated into donor and acceptor fluorescence by a 509 long pass CFP/YFP filter, and then detected by two hybrid photomultiplier tube detectors (Hamamatsu R10467U-40), one having a CFP 483/32 and the other a YFP 537/26 band pass filter for the detection of donor and acceptor signal, respectively. FLIM data were acquired from fields of HEK293 cells using VistaVision software from ISS Inc., and processed by the SimFCS software developed at the Laboratory of Fluorescence Dynamics (LFD), University of California Irvine. The FRET donor fluorophore was excited at 840 nm. An average power of about 5 mW was used to excite the cells. Calibration of the FLIM system was performed by measuring the known lifetime of coumarin 6 in 99% ethanol solution, characterized by a single exponential decay time of 2.55 ns. Typically, the acquisition time to obtain an average photon count of 100 counts/pixel was about 30 sec. Data analysis was performed using the phasor approach to biosensor FLIM-FRET detection^49^. Every pixel of the FLIM image was transformed in one pixel in the phasor plot as previously described and reported in detail^50, 51^. The coordinates *g* and *s* in the phasor plot were calculated from the fluorescence-intensity decay of each pixel of the image by using Fourier transformations. The analysis of the phasor distribution was performed by cluster identification. We calculated the FRET efficiency trajectory according to the classical definition of FRET efficiency:

3 $$E = 1 - \tau DA{\rm{/}}\tau D$$

The phasor of the FRET biosensor in the absence of the activator was obtained from an independent preparation. The phasor corresponding to the quenched donor was calculated according to the quenching equation (3). The positions of all possible phasors that are quenched with different efficiencies describe a curved trajectory in the phasor plot. The experimental position of the phasor of a given pixel along the trajectory determineed the amount of quenching and therefore the FRET efficiency. The contributions of the background and of the donor without acceptor are evaluated using the rule of the linear combination^49, 52^, with the background phasor and the donor unquenched determined independently. All phasor transformations and the data analysis of FLIM data were performed using SimFCS software (University of California, Irvine).

### Electrophysiology

All experimental procedures were carried out in accordance with the National Institutes of Health (NIH) *Guide for the Care and Use of Laboratory Animals* and approved by the University of Southern California Institutional Animal Care and Use Committee. Organotypic slice cultures were prepared from P6–9 male and female Sprague-Dawley rat pups as described in detail in Stoppini et al., 1991^53^. Briefly, hippocampi were removed from P6-P9 Sprague-Dawley rats and 400 μm transverse sections were made using a MX-TS tissue slicer (Siskiyou). Slices were mounted on individual squares of Biopore Membrane filter roll (Millipore) and placed on Millicell Cell Culture inserts (Millipore) in 35 mm dishes containing 1 ml of culture media (MEM + HEPES (Gibco 12360-038), horse serum 25%, HBSS (25%) and L-glutamine (1 mM). Media was exchanged every other day. Sparse biolistic transfections of organotypic slice cultures were performed on DIV1 as described in detail in Schnell et al., 2002^54^. Briefly, 50 µg total of mixed plasmid DNA was coated on 1 µm-diameter gold particles in 0.5 mM spermidine, precipitated in with 0.1 mM CaCl_2_, and washed four times in pure ethanol. The gold particles were coated onto PVC tubing, dried using ultra-pure N_2_ gas, and stored at 4 °C in desiccant. DNA-coated gold particles were delivered with a Helios Gene Gun (BioRad). Construct expression was confirmed by GFP and mCherry epifluorescence. Recordings were performed on day in vitro (DIV) 7 slices. All slices were maintained during recording in room temperature artificial cerebrospinal fluid (aCSF) containing (in mM): 119 NaCl, 2.5 KCl, 1 NaH_2_PO_4_, 26.2 NaHCO_3_, 11 glucose, 4 CaCl_2_ and 4 MgSO_4_. aCSF was supplemented with 5 μM 2-chloroadenosine to dampen epileptiform activity, and GABA_A_ receptors were blocked by picrotoxin (0.1 mM). aCSF was saturated with 95% O_2_/5% CO_2_. The internal whole-cell recording solution contained (in mM): 135 CsMeSO_4_, 8 NaCl, 10 HEPES, 0.3 EGTA, 5 QX-314, 4 Mg-ATP, and 0.3 Na-GTP and pH buffered at 7.3–7.4. Osmolarity was adjusted to 290–295 mOsm.

Transfected neurons were identified by epifluorescence microscopy. All paired recordings involved simultaneous whole-cell recordings from one transfected neuron and a neighboring non-transfected control neuron. Synaptic responses were evoked by stimulating with a monopolar glass electrode filled with aCSF in stratum radiatum of CA1. The stimulus was adjusted to evoke a measurable monosynaptic eEPSC in both cells. Synaptic responses were collected with a Multiclamp 700B amplifier (Axon Instruments, Foster City, CA), filtered at 2 kHz and digitized at 10 kHz. Peak AMPAR currents were recorded at −70 mV. NMDAR currents were measured at + 40 mV and were temporally isolated by measuring amplitudes 150 ms following the stimulus, at which point the AMPAR-eEPSC had completely decayed. In the scatter plots for simultaneous dual recordings, each open circle represents one paired recording, and the closed circle represents the average of all paired recordings. In the scatter plot, the x-axis represents the eEPSC recorded in the control cell, and the *y* axis represents the eEPSC recorded in the transfected cell. Virtual 1:1 diagonal line is also shown. If the data point falls below the diagonal line, it indicates that the eEPSC is higher in the control cell. Paired-pulse ratio was determined by delivering two stimuli at 40 ms apart and dividing the peak response of stimulus 2 by the peak response to stimulus 1. Series resistance was monitored and not compensated, and neurons in which series resistance varied by 25% during a recording session were discarded. No more than one paired recording was performed on a given slice. Data was collected and analyzed using in-house software in Igor Pro (Wavemetrics) developed in Dr. Roger Nicoll’s laboratory at UCSF.

### Spine density analysis

For spine density analysis, control and experimental CA1 pyramidal neurons in organotypic hippocampal slice cultures made from P6 rat pups were biolistically transfected with FUGW-GFP and pCAGGS-IRES-mCherry constructs approximately 18–20 h after plating. Images were acquired at DIV 7 using super-resolution microscopy (Elyra Microscope System, Zeiss). For use with the available inverted microscope and oil-immersion objective lens, slices were fixed in 4% PFA/4% sucrose in PBS and washed 3× with PBS. To amplify the GFP signal, slices were then blocked and permeabalized in 3% BSA in PBS containing 0.1% Triton-X and stained with primary antibody against GFP (2 μg/ml, Life Technologies A-11122) followed by washes in PBSTx and staining with Alexa 488-conjugated secondary (4 μg/ml, Life Technologies A-11034). Slices were further processed with an abbreviated SeeDB-based protocol^55^ in an attempt to reduce spherical aberration. Slices were then mounted in SlowFade Gold (Life Technologies) for imaging. Z-stacks were made of 30 µm sections of secondary apical dendrites ~30 µm from the soma. Images were acquired with a 100× oil objective (100×/1.46) in SIM mode using a supplied 42 μm SIM grating and processed and reconstructed using supplied software (Zen, Zeiss). An experimenter, blinded to the condition of the image, performed image analysis on individual sections using ImageJ to count spines extending laterally from the dendrite.

### Statistics analysis

For the ASD-related mutation statistics in Trio, we used a simple Poisson test, similar to the one used in Samocha et al.^29^. The expected number of de novo mutations in Trio protein in 4890 individuals with ASD-related disorders was calculated based on gene-specific mutation probability as determined by Samocha et al. Expected number of de novo mutations in the Trio-GEF1/DH1 domain was estimated through rescaling of expected number of mutations in Trio with assumption of equal mutation probability along the gene. A genome-wide significance threshold *P*-value of < 1 × 10^−6^ was used. For paired electrophysiological recordings of eEPSC amplitude, a Wilcoxon Signed Rank Test for paired data was used. Wilcoxon Rank Sum Tests were used to compare electrophysiological data across independent conditions. Paired pulse facilitation measurements and spine density data were analyzed using a paired and unpaired Student’s *t*-test, respectively. All statistical tests performed were two-sided and with all tests a *P*-value of < 0.05 was considered statistically significant. All error bars represent standard error measurement. Sample sizes in the present study are similar to those reported in the literature.

### Data availability

All data are available from the authors upon request.

## Electronic supplementary material

Supplementary Information
