# Supplementary material for: An autism spectrum disorder-related de novo mutation hotspot discovered in the GEF1 domain of Trio
Source: Nat Commun. 2017 Sep 19;8:601. doi: 10.1038/s41467-017-00472-0 (PMC5605661; doi:10.1038/s41467-017-00472-0)
Supplement: Supplementary file 1 — Supplementary Information [file 41467_2017_472_MOESM1_ESM.pdf]

File name: Supplementary Information
Description: Supplementary Figures, Supplementary Tables and Supplementary References

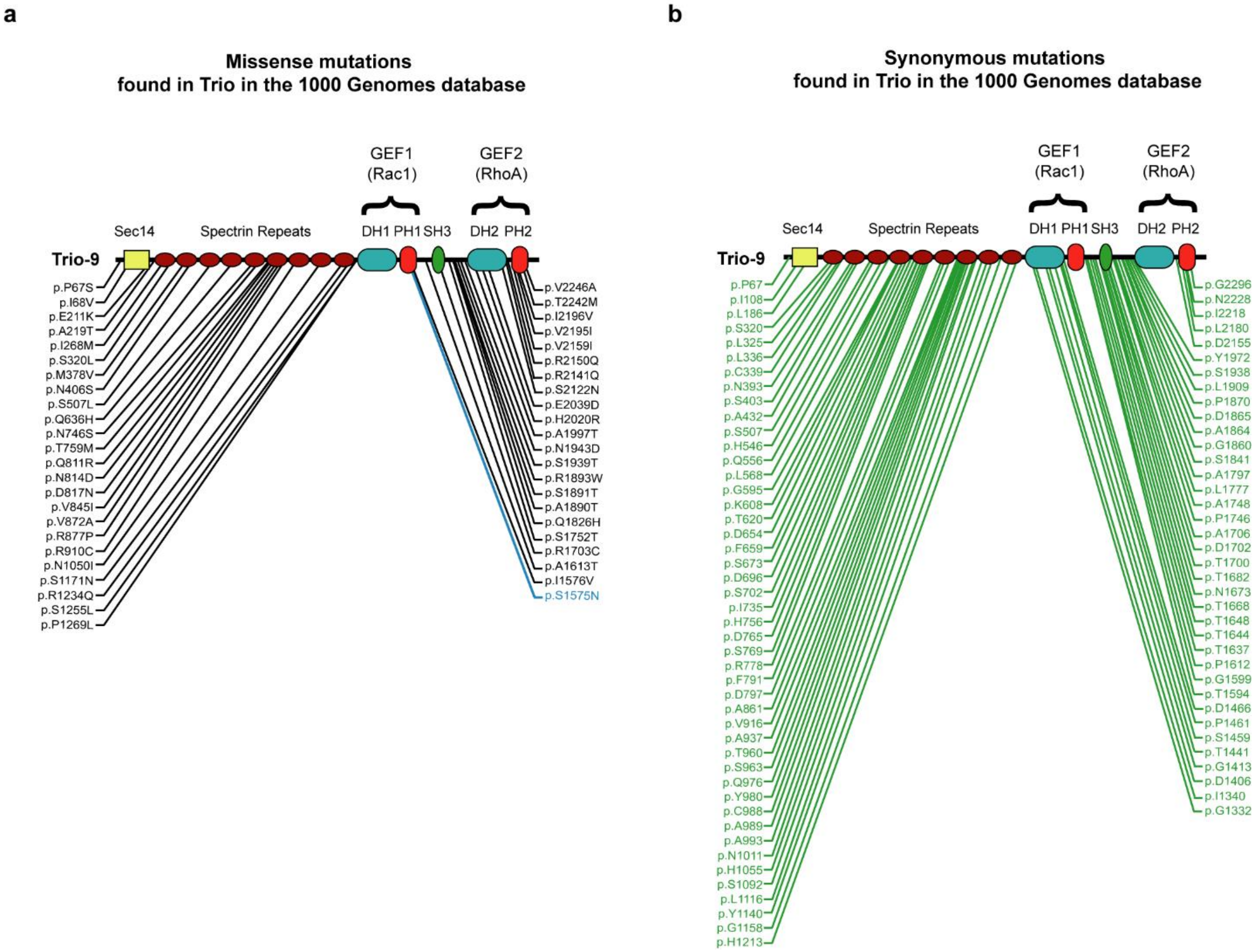

Supplementary Figure 1. Missense and synonymous mutations found in Trio in the 1000 genomes database **(a)** Illustration of the positions of all missense Trio mutations found in individuals in the 1000 Genomes database[1]. Trio-9 S1575N (in blue) was tested for an effect on Trio-9 function (Supplementary Fig. 5) **(b)** Illustration of the positions of all synonymous Trio mutations found in individuals in the 1000 Genomes database.

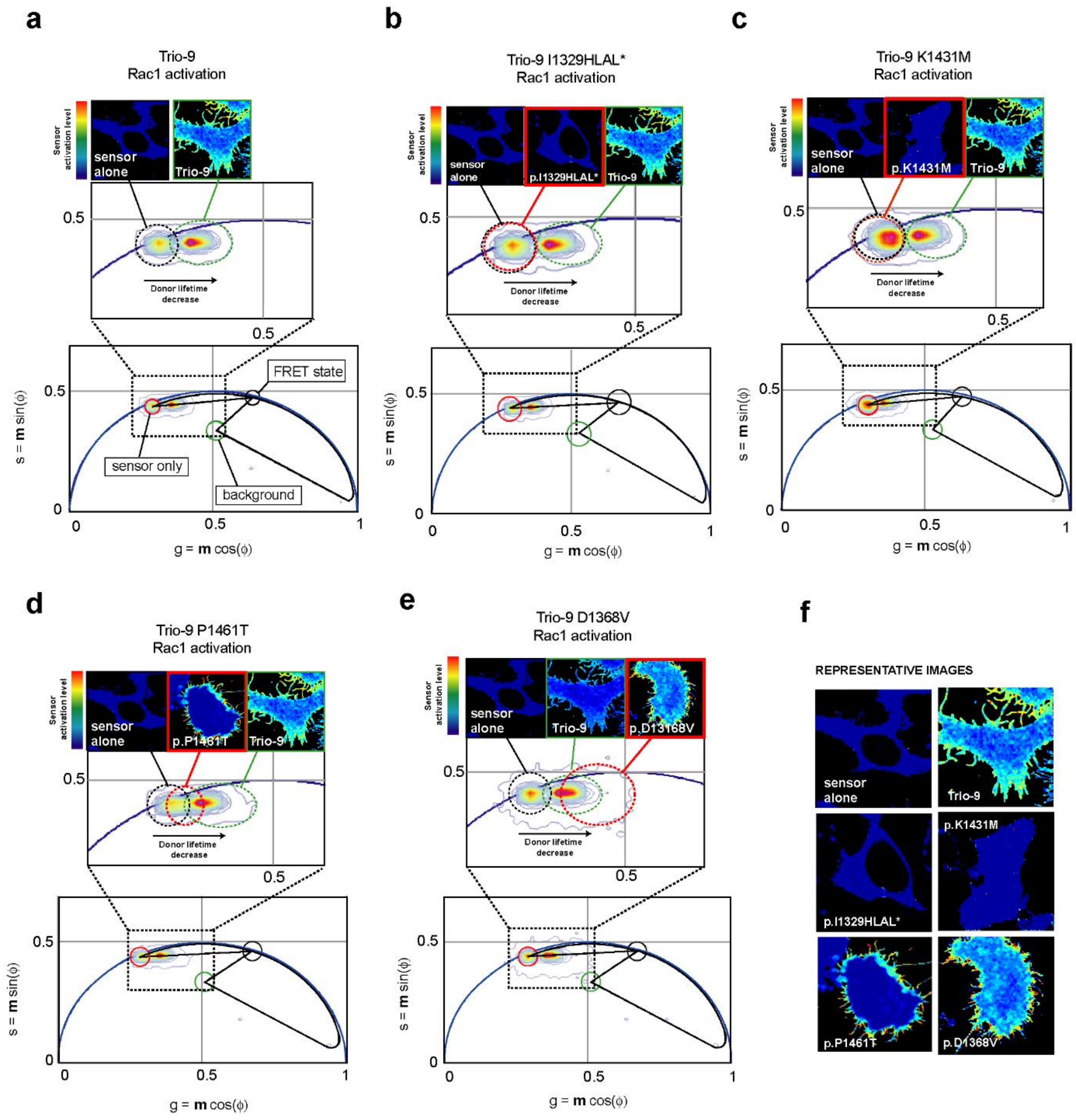

Supplementary Figure 2. FLIM-based FRET analysis of ASD-related de novo mutations in Trio. (a-e) Shown above are representative FLIM color maps of HEK293 cells expressing the Rac1 biosensor alone and the biosensor together with the versions of Trio-9 tested. Colors range from blue to red with increasing biosensor activation. A cropped and enlarged region of the phasor plot is shown in the middle for HEK293 cells transfected with the biosensor alone and the biosensor with the versions of Trio-9 tested. Dashed ovals identify the phasor distribution for each experimental condition. Whole phasor plots shown below, the cluster indicated by the solid red circle represents HEK293 cells expressing only the Rac1 biosensor, the solid black circle represents the on state of the biosensor and the solid green circle represents the background fluorescence. The theoretical FRET trajectory (black curve) is superimposed with the black line passing through the phasor distributions to determine the biosensor FRET efficiency. FLIM analysis is shown for **(a)** Trio-9 **(b)** Trio-9 I1329HLAL*, **(c)** Trio-9 K1431M, **(d)** Trio-9 P1461T and **(e)** Trio-9 D1368V. **(f)** Enlarged representative FLIM color maps for each condition.

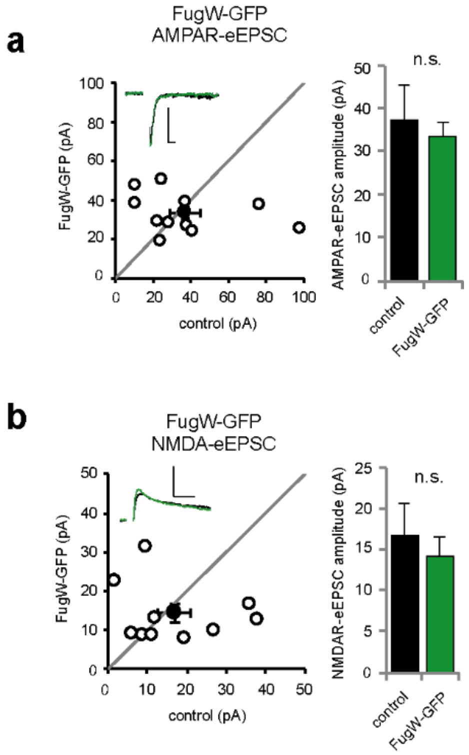

Supplementary Figure 3. Strong fluorophore expression does not affect glutamatergic synaptic transmission at Schaffer collateral synapses. (a-b) Scatterplots show AMPAR and NMDAR-eEPSC amplitudes for single pairs of control and GFP transfected CA1 pyramidal neurons (open circles). Filled circles show mean ± SEM. (Insets) Sample current traces from control (black) and transfected (green) neurons (Scale bars: 20 ms for AMPA, 50 ms for NMDA, 20 pA). Bar graphs show the average eEPSC amplitudes (± SEM) of untransfected and GFP transfected neurons. **(a)** In CA1 pyramidal neurons GFP expression using a ubiquitin promoter does not affect AMPAR-eEPSC amplitude relative to untransfected control neurons (n = 11 pairs, $p > 0.05$, Wilcoxon Sign Rank Test). **(b)** In CA1 pyramidal neurons GFP expression using a ubiquitin promoter does not affect NMDAR-eEPSC amplitude relative to untransfected control neurons (n = 10 pairs, $p > 0.05$, Wilcoxon Signed Rank Test). n.s., not significant.

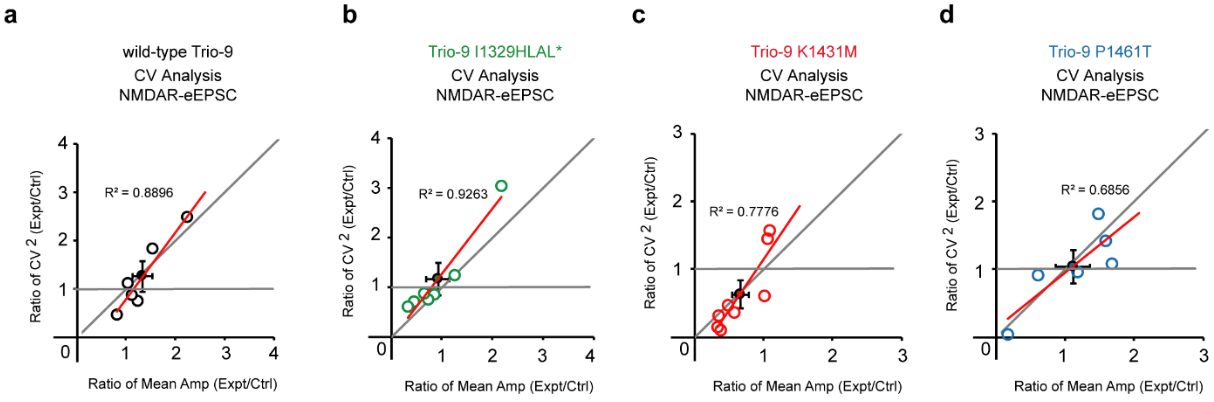

Supplementary Figure 4. CV analysis of NMDAR-eEPSCs in neurons expressing Trio-9, Trio-9 p.I1329HLAL*, Trio-9 p.K1431M and Trio-9 p.P1461T. (a-d) Scatter plots show CV^{-2} graphed against ratio of mean amplitude within each pair (open circles). Filled circles show mean ± SEM. Linear regression analysis is performed on the data and the R^2 value of the regression line is provided in the graphs. **(a)** CV analysis of NMDAR-eEPSCs from control and Trio-9 expressing neurons indicates that NMDAR-eEPSC amplitude variability between pairs of neurons stems from variability in quantal content. **(b)** CV analysis of NMDAR-eEPSCs from control and Trio-9 I1329HLAL* expressing neurons indicates that NMDAR-eEPSC amplitude variability between pairs of neurons stems from variability in quantal content. **(c)** CV analysis of NMDAR-eEPSCs from control and K1431M expressing neurons indicates that NMDAR-eEPSC amplitude variability between pairs of neurons stems from variability in quantal content. **(d)** CV analysis of NMDAR-eEPSCs from control and P1461T expressing neurons indicates that NMDAR-eEPSC amplitude variability between pairs of neurons stems from variability in quantal content.

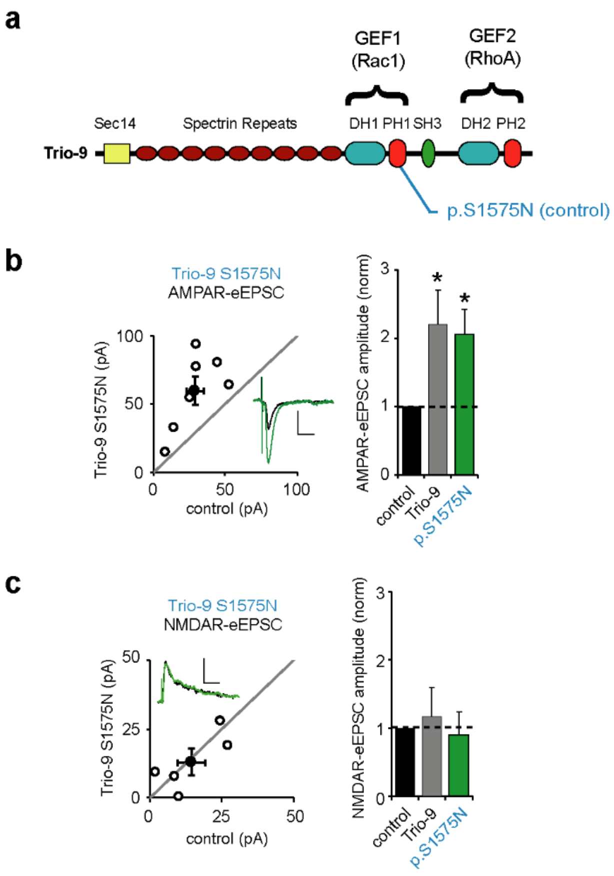

Supplementary Figure 5. Trio-9 p.S1575N does not alter Trio-9-mediated potentiation of synaptic AMPAR expression. (a) Protein domain illustration of Trio-9 S1575N. **(b-c)** Scatterplots to the left show amplitudes of AMPAR and NMDAR-eEPSCs for single pairs of control and transfected neurons (open circles). Filled circles show mean ± SEM. (Insets) Sample current traces from control (black) and transfected (green) neurons (Scale bars: 20 ms for AMPA, 50 ms for NMDA, 20 pA). Bar graphs show the average eEPSC amplitudes (± SEM) of neurons expressing Trio-9 (from Fig. 4j,k) and Trio-9 S1575N normalized to the corresponding average control neuron eEPSC amplitude. **(b)** Trio-9 S1575N expression in CA1 pyramidal neurons causes an increase in AMPAR-eEPSC amplitude relative to untransfected control neurons ($n = 7$ pairs, $^*p < 0.05$, Wilcoxon Sign Rank Test). **(c)** Trio-9 S1575N expression in CA1 pyramidal neurons did not affect NMDAR-eEPSC amplitude ($n = 5$ pairs, $p > 0.05$, Wilcoxon Sign Rank Test).

Supplementary Table 1 | Summary of putative etiologic sequence variants in TRIO

	Mutation (DNA level)	Mutation (protein level)	Protein domain	Disorder	Supplementary Reference number
	c.G2472T	p.E883D	SR6	Autism	2
	c.A3062T	p.N1080I	SR8	NDD,ID	3
	c.C3757T	p.R1312W	GEF1	Autism	4
	c.A3926T	p.D1368V	GEF1	ID	5
	c.G4106A	p.R1428Q	GEF1	NDD,ID	3
	c.A4115T	p.K1431M	GEF1	ASD,ID	6
	c.C4204A	p.P1461T	GEF1	NDD	3
	c.C4205T	p.P1461L	GEF1	NDD	3
	CNV at 5:14380988-14463257(del)	p.1149-1889 (deleted)	SR9, GEF1, SH3	NDD	3
	c.T3809_	p.I1329HLAL*	GEF1, GEF2	ASD	7
	c.G6481C	p.V2220L	GEF2 (PH2)	ASD	7
REF:	NM_007118.2 or Human (GRCh37)	NP_009049.2			

Supplementary Table 2 | Number of de novo mutations found in known ASD-risk genes in 4890 individuals with ASD-related disorders

GENE	Number of Mutations	cDNA Length (base pairs)	ASD Mutations Rate per base pair
SCN2A	22	6018	3.66×10^{-3}
SYNGAP	15	4032	3.72×10^{-3}
TRIO	**11**	**9294**	$\mathbf{1.18 \times 10^{-3}}$
TRIO-GEF1/DH1	**7**	**525**	$\mathbf{1.33 \times 10^{-2}}$
SHANK2	4	5553	7.20×10^{-4}
NRXN1	4	4644	8.61×10^{-4}
MECP2	3	1497	2.0×10^{-3}
CNTNAP4	3	3708	8.09×10^{-4}
SNANK3	2	5244	3.81×10^{-4}
KALRN	0	4992	0

Supplementary Table 3 | Significance of *TRIO and TRIO-GEF/DH1* mutations in ASD-related disorders

	TRIO	***TRIO-GEF1/DH1***
Length, base pairs	9294	525
Expected rate of DNM per gene	2.19×10^{-4}	1.23×10^{-5}
Expected number of DNM per gene in 4890 individuals with ASD-related disorders	1.07	0.060
Observed number of DNM per gene in 4890 individuals with ASD-related disorders	11	7
P-value	1.96×10^{-8}	5.50×10^{-13}

Supplementary References

1. Genomes Project, C. *et al.* A global reference for human genetic variation. *Nature* **526**, 68-74 (2015).
2. Iossifov, I. *et al.* The contribution of de novo coding mutations to autism spectrum disorder. *Nature* **515**, 216-221 (2014).
3. Deciphering Developmental Disorders, S. Large-scale discovery of novel genetic causes of developmental disorders. *Nature* **519**, 223-228 (2015).
4. O'Roak, B.J. *et al.* Sporadic autism exomes reveal a highly interconnected protein network of de novo mutations. *Nature* **485**, 246-250 (2012).
5. de Ligt, J. *et al.* Diagnostic exome sequencing in persons with severe intellectual disability. *N Engl J Med* **367**, 1921-1929 (2012).
6. Sanders, S.J. *et al.* De novo mutations revealed by whole-exome sequencing are strongly associated with autism. *Nature* **485**, 237-241 (2012).
7. De Rubeis, S. *et al.* Synaptic, transcriptional and chromatin genes disrupted in autism. *Nature* **515**, 209-215 (2014).
